# Supplementary material for: Bi-enzymatic chemo-mechanical feedback loop for continuous self-sustained actuation of conducting polymers
Source: Nat Commun. 2023 Oct 12;14:6390. doi: 10.1038/s41467-023-42153-1 (PMC10570360; doi:10.1038/s41467-023-42153-1)
Supplement: Supplementary file 1 — Supplementary information [file 41467_2023_42153_MOESM1_ESM.docx]

Supplementary Information

Bi-enzymatic chemo-mechanical feedback loop for continuous self-sustained actuation of conducting polymers

Serena Arnaboldi^1†^, Gerardo Salinas^2†^, Sabrina Bichon^3^, Sebastien Gounel^3^, Nicolas Mano^3^, Alexander Kuhn^2^*

^1^Dip. Di Chimica, Univ. degli Studi di Milano, Milan, Italy

^2^Univ. Bordeaux, CNRS, Bordeaux INP, ISM, UMR 5255, F-33607 Pessac, France

^3^Centre de Recherche Paul Pascal, Univ. Bordeaux, CNRS, UMR 5031, Pessac, France

*Corresponding author contact: [kuhn@enscbp.fr](mailto:kuhn@enscbp.fr)

^†^These authors contributed equally

**Supplementary Methods**

**Polypyrrole enzymatic modification.** Both anodic (glucose oxidase from *A. niger*) and cathodic (Bilirubin oxidase from *M. oryzae*) enzymes have been produced and purified as follows^1,2^.

*Glucose oxidase from A.niger.* Glucose Oxidase (GOx) from *Aspergilus niger* (E.C. 1.1.3.4) was purchased from Sigma Aldrich and was first purified by hydrophobic interaction chromatography and then by anion exchange chromatography using an AKTA purifier system. After dissolving the GOx in a 20mM sodium phosphate buffer pH 7.5 (NaH_2_PO_4_, Sigma-Aldrich, ≥99.0%, and Na_2_HPO_4_, Sigma-Aldrich, ≥99.0%) containing 1M (NH_4_)_2_SO_4_ (Sigma-Aldrich, ≥99.0%) the enzyme is loaded onto a 1 mL HiTrap Phenyl HP column pre-equilibrated with the binding buffer. The solution is then eluted at 1 ml/min with a linear gradient of 1–0M (NH_4_)_2_SO_4_ in a 20mM sodium phosphate buffer, pH 7.5 and continuously monitored at 280nm and 452nm respectively, to discriminate protein and its cofactor FAD (Flavin Adenine Dinucleotide). The active fractions were then pooled, concentrated with an Amicon Ultra 15 membrane (cut off 10 kDa) and desalted on a PD10 column with the binding buffer used for the second step (20mM sodium phosphate buffer, pH 8.0). GOx solution was then applied to a HiTrap Q FF column (1ml) pre-equilibrated with 20mM sodium phosphate buffer, pH 8.0. The solution was then eluted at 1ml/min with a linear gradient of 0–0.4M NaCl (Sigma-Aldrich, ≥99.5%) in the same phosphate buffer, pH 8.0. Fractions containing GOx activity were pooled, concentrated and equilibrated with 30mM sodium phosphate, pH 5.1 and stored at 4 °C. The homogeneity of the purified GOx was checked by SDS-PAGE (10%) followed by Coomassie blue staining. To confirm that 2 FADs were bound per protein, the protein and FAD concentrations were calculated from its absorption at 460 nm (ε = 12.83 mM^-1^ cm^-1^) and the protein concentration by its absorption at 280 nm (ε = 263 mM^-1^ cm^-1^). A standard oxidase assay using HRP and ABTS was used to determine enzyme activity while considering GOx as a dimer since only the dimeric form is active. Steady-state kinetics (k_ss_) were followed by continuous monitoring of adsorption at 410 nm (ε = 36.8 mM^-1^ cm^-1^). The GOx solution was added to a 3 mL reaction mixture containing 16 mM ABTS, 1.9 U mL^-1^ HRP and 167 mM glucose (Sigma-Aldrich, ≥99.5%) in 100 mM sodium acetate (Sigma-Aldrich, ≥99.0%), pH 6.0. Measurements were performed at 25 °C, pH 6.0, under air-saturated conditions.

*Bilirubin Oxidase from M. Oryzae.* Hydrophobic interaction chromatography using an AKTA purifier system was used to purify the BOD in a single step. After concentration by ultrafiltration with a 10-kDa YM10 membrane, 5 mL of the culture media were first equilibrated in a 50-mM potassium phosphate (Sigma-Aldrich, ≥99.0%), 1.7 M (NH_4_)_2_SO_4_ pH 6, and then injected on the HiLoad 26/10 Phenyl Sepharose HP equilibrated in the same buffer. BOD was eluted at a flow rate of 2.5 ml min^−1^ with a linear gradient of 1.7 to 0M (NH_4_)_2_SO_4_ in a 50-mM potassium phosphate buffer (KH_2_PO_4_, Sigma-Aldrich, ≥99.0%, and K_2_HPO_4_, Sigma-Aldrich, ≥99.0%), pH 6. Protein elution was monitored at 280 nm and 600 nm to distinguish between impurities and BOD. Active fractions were pooled, concentrated using an Amicon Ultra 15 membrane (cut-off, 10 kDa), and desalted on a PD10 column with a 50mM potassium phosphate buffer, pH 6. The BOD was then stored at 0 °C. Enzyme activity was determined spectrophotometrically at 37 °C by following the oxidation of 1 mM ABTS at 410 nm (ε =36.8 mM^−1^ cm^−1^) in a Mcllvaine's citrate/phosphate buffer, pH 4.

The anodic hydrogel was prepared by mixing 43.5 μl of anodic Os-redox polymer (PVP-Os[(1,1-dimethyl-2,2′biimidazole)2-2-[6-methylpyrid-2yl] imidazole]^2+/3+^; 8.0 mg ml^-1^ in MilliQ water) with 43.3 μl of glucose oxidase solution (5.0 mg ml^-1^ in 0.3 M phosphate buffer solution pH 5) and 30.9 μl of poly(ethylene glycol) diglycidyl ether (PEGDGE, Sigma-Aldrich) as a cross-linker (2.0 mg ml^-1^ in MilliQ water), respectively. The wt% ratio of the GOx/Os-polymer/PEGDGE in the anodic hydrogel was 35/55/10. 106.2 μl of the so-obtained hydrogel were carefully positioned at the surface of a free-standing Ppy film (1.5 cm x 1.5 cm area) and dried at 4 °C for 48h. Total loading = 250 μg cm^-2^.

For the preparation of the cathodic composite hydrogel, 80.6 μl of the cathodic Os-redox polymer (PAA-PVI-[Os(4,4′-dichloro-2,2′-bipyridine)_2_Cl]^+/2+^; 10 mg mL^−1^ in MilliQ water) was mixed with 38.6 μl of BOD and 23.9 μl of PEGDGE cross-linker, respectively. 198.6 μl of this composite suspension were positioned at the surface of a free-standing Ppy film (1.5 cm x 1.5 cm area) and dried at 4 °C for 48h. The ratio between BOD/Os-polymer/PEGDGE was kept at 30/62.57/7.43. Total loading = 416.7 μg cm^-2^.

**Electrochemical measurements**

Electrochemical characterization of the modified anodic and cathodic sides of the actuators were performed using a CHI bipotentiostat in a classic three-electrode set up in a 0.3 M PBS buffer solution at 37.5 °C (pH = 5) saturated with Ar at 10 mV s^-1^. Electropolymerization and characterization studies of Ppy were performed in a classic three-electrode cell containing a 0.1 M tetra butyl ammonium perchlorate/acetonitrile (TBAP, Sigma-Aldrich, ≥99.5% /ACN, Sigma-Aldrich, anhydrous, 99.8%) solution at 25°C and 10 mV s^-1^, by means of a PalmSense4 potentiostat, connected to a personal computer. Potentiodynamic polymerization was carried out in a 5 mM pyrrole monomer solution. The working electrode was in this case a 3 mm diameter glassy carbon disk electrode, whereas Pt and Ag wires were used as the counter and pseudo reference electrodes, respectively. All the potential values are referenced by conversion to a Ag/AgCl electrode and the normalized current was calculated as the ratio between the current at each potential and the maximum current. Double pulse potentiostatic measurements at 0.8 V and -0.8 V were carried out in a three-electrode cell containing a 0.1 M LiClO_4_ aqueous solution at 25°C, using a free-standing Ppy/DBS film as working electrode, and Pt and Ag/AgCl as the counter and reference electrodes, respectively.

**Wireless asymmetric modification**

For the asymmetric modification by bipolar electrochemistry, pristine Ppy strips (0.5 cm x 1.5 cm) were placed in the center of a bipolar cell. Two graphite feeder electrodes were positioned at the extremities of the cell (5 cm apart). An aqueous 0.2 M LiClO_4_ solution was used as supporting electrolyte to provide a sufficient amount of ions for charge compensation in the conducting polymer during the bipolar modification.

**Polypyrrole resistance measurements**

The resistance of the Ppy/DBS films was measured by means of a digital multimeter. Resistance measurements were carried out before and after the wireless asymmetric polarization of the polymer, in six different regions along the Ppy film.


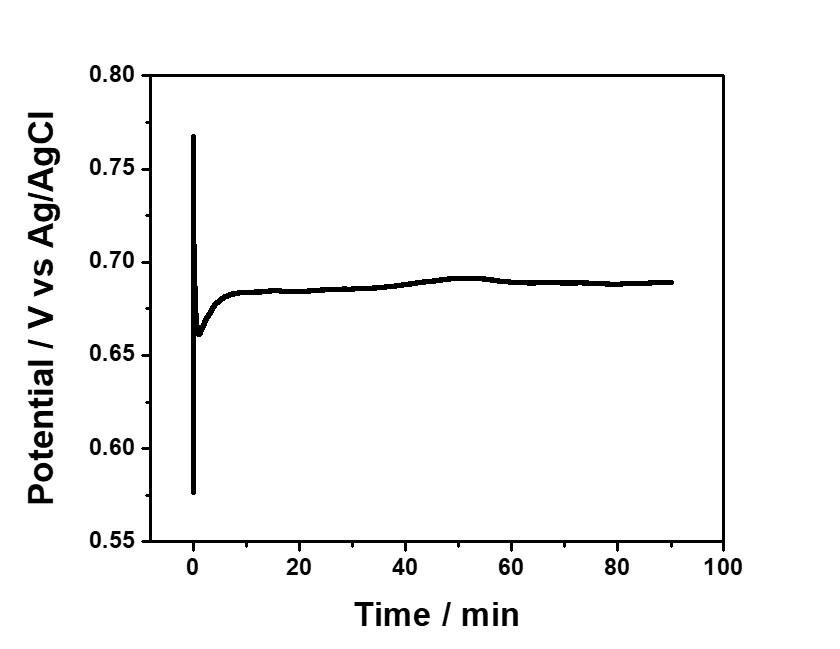


**Supplementary Figure 1. Electropolymerization of pyrrole.** Potential transient plot obtained by applying a constant current of 4 mA for 1.5 h to a gold-coated glass slide dipped in a 0.2 M pyrrole, 0.25 M sodium dodecylbenzenesulfonate (DBS) aqueous solution.


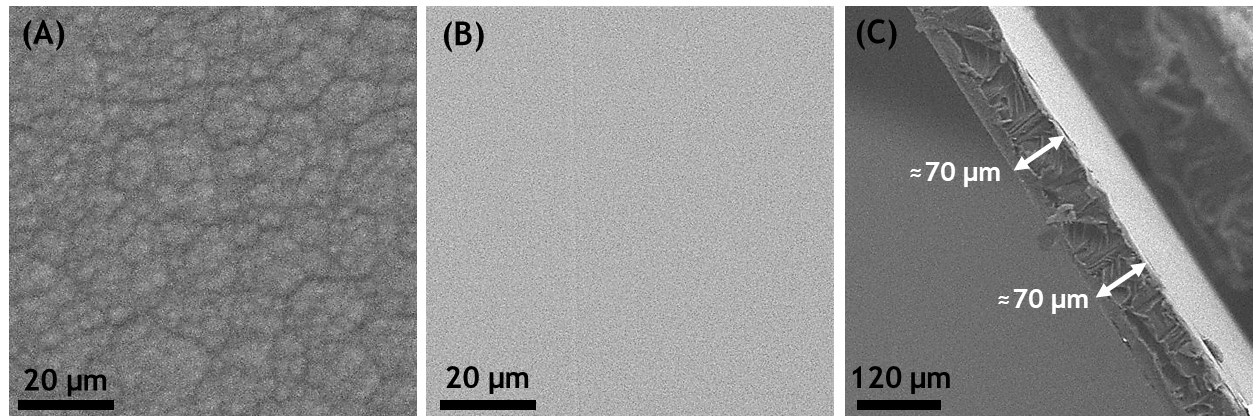


**Supplementary Figure 2. SEM micrographs of the freestanding Ppy film.** SEM image of the (A) rough and (B) smooth face of a freestanding Ppy film. (C) SEM image of the cross-section of a typical Ppy film.


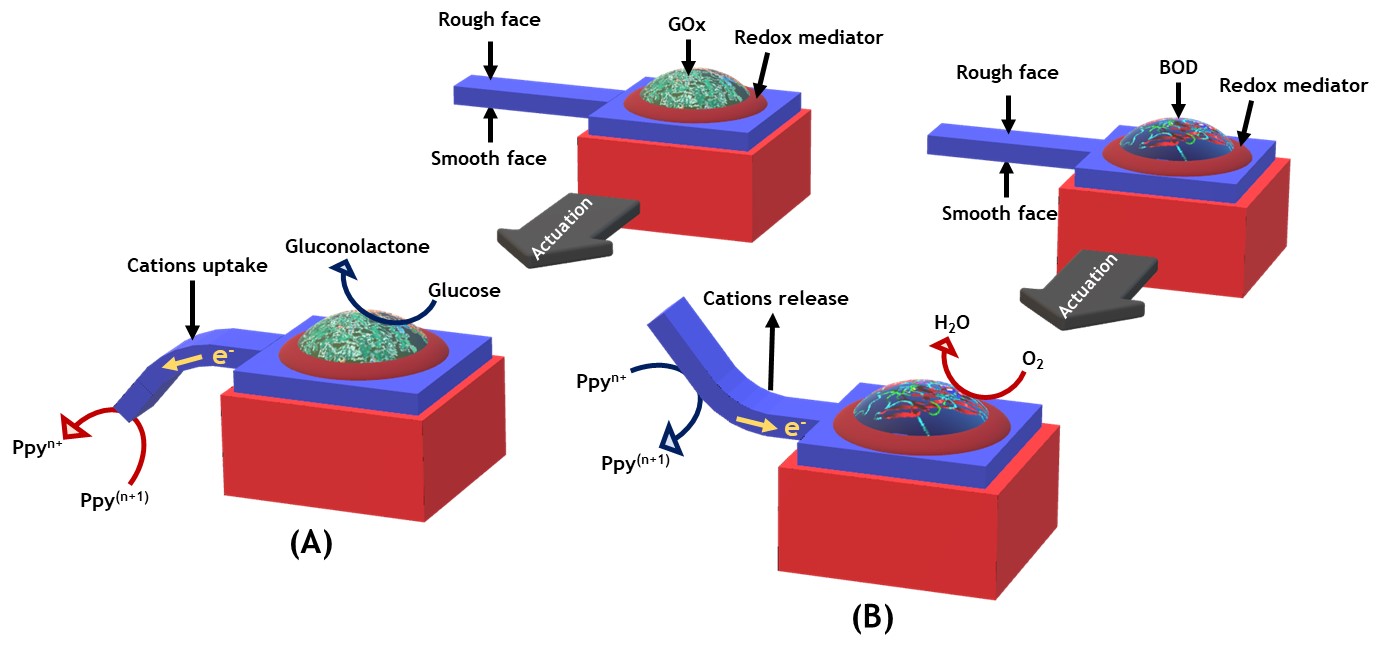


**Supplementary Figure 3. Mono-enzymatic bioelectrochemical actuation**. Illustration of the autonomous bending of a bioelectrochemical actuator driven by (A) GOx and (B) BOD, before and after the corresponding bending, with a representation of the associated reactions. The red ring symbolizes the redox polymer and the cross-linker, whereas the blue part symbolizes the Ppy/DBS film.


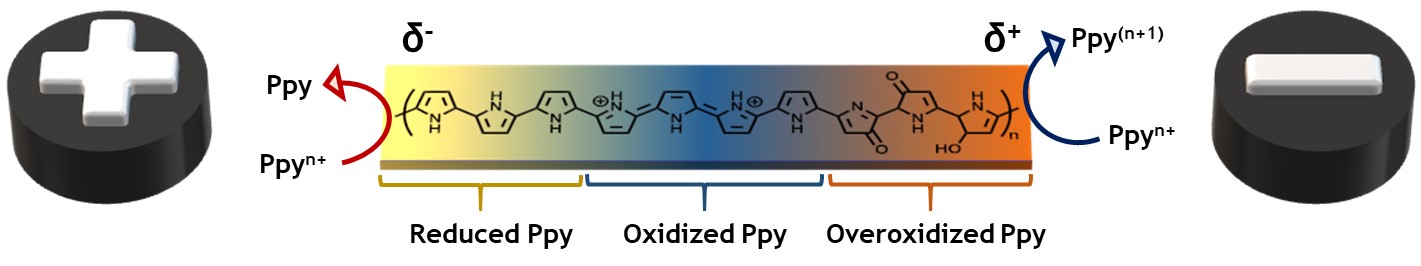


**Supplementary Figure 4. Asymmetric modification by bipolar electrochemistry**. Illustration of the wireless modification of a charged Ppy/DBS strip placed between two feeder electrodes, with a representation of the chemical structures and the associated redox reactions. The different colors symbolize the three main redox regions of the polymer; reduced Ppy (yellow)), oxidized Ppy (blue), and overoxidized Ppy (orange).


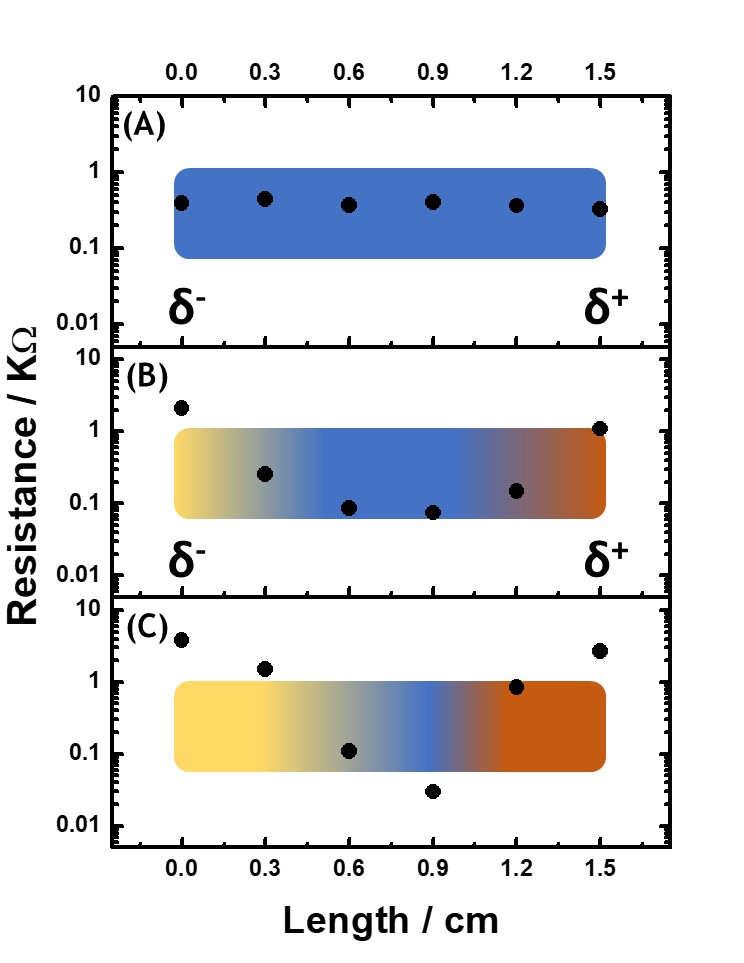


**Supplementary Figure 5. Resistance measurements.** Resistance values at different positions along the Ppy film, for (A) pristine Ppy and asymmetrical modified Ppy strips obtained by applying an electric field of 4 Vcm^-1^ for (B) 10 seconds and (C) 20 seconds in a bipolar electrochemical preconditioning experiment. The different colors symbolize the three main redox regions of the polymer; reduced Ppy (yellow)), oxidized Ppy (blue) and overoxidized Ppy (orange).


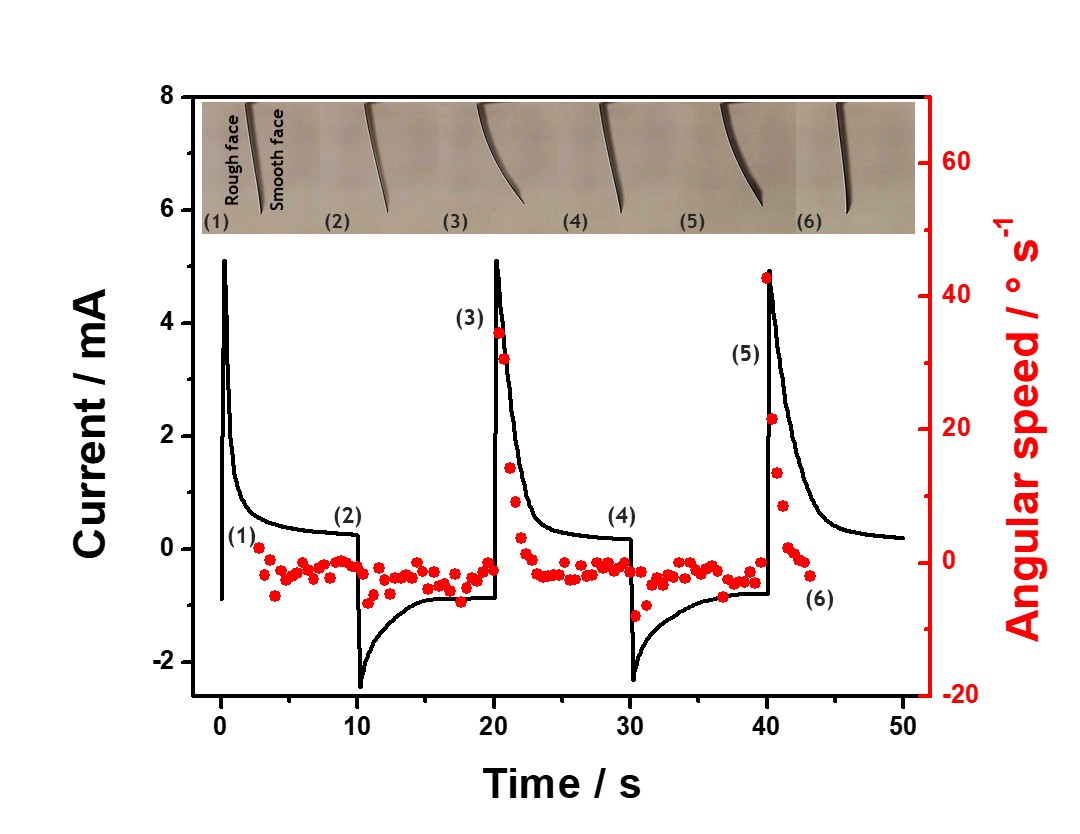


**Supplementary Figure 6. Double pulse potentiostatic measurements.** Double pulse current (black curve) and angular speed transients (red dots), recorded with a free-standing Ppy/DBS film in a 0.1 M LiClO_4_ solution; oxidation potential 0.8 V vs Ag/AgCl, reduction potential -0.8 V vs Ag/AgCl and pulse time 10 seconds. The inset shows the corresponding pictures of the actuation at different times.

**Supplementary References**

1. O. Courjean, N. Mano, Recombinant glucose oxidase from Penicillium amagasakiense for efficient bioelectrochemical applications in physiological conditions. *J. Biotechnol.* **151**, 122-9, (2011).

2. F. Durand, S. Gounel, C. H. Kjaergaard, E. I. Solomon, N. Mano, Bilirubin oxidase from Magnaporthe oryzae: An attractive new enzyme for biotechnological applications. *Appl. Microbiol. Biotechnol*. **96**, 1489-1498, (2012).
