# Supplementary material for: Bi-enzymatic chemo-mechanical feedback loop for continuous self-sustained actuation of conducting polymers
Source: Nat Commun. 2023 Oct 12;14:6390. doi: 10.1038/s41467-023-42153-1 (PMC10570360; doi:10.1038/s41467-023-42153-1)
Supplement: Supplementary file 3 — Description of Additional Supplementary Files [file 41467_2023_42153_MOESM3_ESM.pdf]

Description of Additional Supplementary Files

File Name: Supplementary Movie 1
Description: **Mono-enzymatic actuation measurements (real time).** Autonomous bioelectrochemical actuation of (left side) a Ppy/DBS/GOx film in a 0.3 M PBS (pH = 5), 1 mM glucose solution and (right side) a Ppy/DBS/BOD film in a 0.3 M PBS solution at 25°C (pH = 5) saturated with O_2.

File Name: Supplementary Movie 2
Description: **Bi-enzymatic actuation as a function of glucose concentration (x2 accelerated).** Self-sustained enzymatic electromechanical actuation as a function of glucose concentration of a modified bi-enzymatic Ppy/DBS films in a 0.3 M PBS solution at 25°C (pH = 5) saturated with O_2.

File Name: Supplementary Movie 3
Description: **Lateral motion of an asymmetrically modified bioelectrochemical actuator strip 1 (x5 accelerated)**. Lateral motion of an asymmetrically modified Ppy/DBS film immersed in a 0.3 M PBS at 25°C (pH = 5), 5 mM glucose solution, saturated with O_2.

File Name: Supplementary Movie 4
Description: **Lateral motion of an asymmetrically modified bioelectrochemical actuator strip 2 (x5 accelerated)**. Lateral motion of an asymmetrically modified Ppy/DBS film with a shorter region of electroactive Ppy and an inverted orientation immersed in a 0.3 M PBS at 25°C (pH = 5), 5 mM glucose solution, saturated with O_2.

File Name: Supplementary Movie 5
Description: **Double pulse potentiostatic actuation of Ppy (x10 accelerated)**. Electromechanical actuation of a free-standing Ppy/DBS film in a 0.1 M $LiClO_4$ solution; oxidation potential 0.8 V vs Ag/AgCl, reduction potential -0.8 V vs Ag/AgCl and pulse time 10 seconds.

File Name: Supplementary Movie 6
Description: **Long distance lateral motion of an asymmetrically modified bioelectrochemical actuator strip (x10 accelerated)**
Top view of a long distance motion of an asymmetrically modified Ppy/DBS film with a short region of electroactive Ppy in a 0.3 M PBS at 25°C (pH = 5), 5 mM glucose solution, saturated with O_2.
